# Identification of LncRNAs Associated With FOLFOX Chemoresistance in mCRC and Construction of a Predictive Model

**DOI:** 10.3389/fcell.2020.609832

**Published:** 2021-01-28

**Authors:** Yiyi Zhang, Meifang Xu, Yanwu Sun, Ying Chen, Pan Chi, Zongbin Xu, Xingrong Lu

**Affiliations:** ^1^Department of Colorectal Surgery, Fujian Medical University Union Hospital, Fuzhou, China; ^2^Department of Pathology, Fujian Medical University Union Hospital, Fuzhou, China; ^3^Department of Plastic Surgery, Fuzhou Dermatosis Prevention Hospital, Fuzhou, China

**Keywords:** colorectal cancer, FOLFOX, Gene Array Chip, WGCNA, lncRNA

## Abstract

Oxaliplatin, fluorouracil plus leucovorin (FOLFOX) regimen is the first-line chemotherapy of patients with metastatic colorectal cancer (mCRC). However, studies are limited regarding long non-coding RNAs (lncRNAs) associated with FOLFOX chemotherapy response and prognosis. This study aimed to identify lncRNAs associated with FOLFOX chemotherapy response and prognosis in mCRC patients and to construct a predictive model. We analyzed lncRNA expression in 11 mCRC patients treated with FOLFOX chemotherapy before surgery (four sensitive, seven resistant) by Gene Array Chip. The top eight lncRNAs (AC007193.8, CTD-2008N3.1, FLJ36777, RP11-509J21.4, RP3-508I15.20, LOC100130950, RP5-1042K10.13, and LINC00476) for chemotherapy response were identified according to weighted correlation network analysis (WGCNA). A competitive endogenous RNA (ceRNA) network was then constructed. The crucial functions of the eight lncRNAs enriched in chemotherapy resistance were mitogen-activated protein kinase (MAPK) and proteoglycans signaling pathway. Receiver operating characteristic (ROC) analysis demonstrated that the eight lncRNAs were potent predictors for chemotherapy resistance of mCRC patients. To further identify a signature model lncRNA chemotherapy response and prognosis, the validation set consisted of 196 CRC patients from our center was used to validate lncRNAs expression and prognosis by quantitative PCR (qPCR). The expression of the eight lncRNAs expression between CRC cancerous and adjacent non-cancerous tissues was also verified in the validation data set to determine the prognostic value. A generalized linear model was established to predict the probability of chemotherapy resistance and survival. Our findings showed that the eight-lncRNA signature may be a novel biomarker for the prediction of FOLFOX chemotherapy response and prognosis of mCRC patients.

## Introduction

Colorectal cancer (CRC), common cancer, is the second leading cause of cancer-related death in the world (Edwards et al., 2014). Chemotherapy has been widely used in the treatment of mCRC patients. Oxaliplatin, fluorouracil plus leucovorin (FOLFOX) regimen is the first-line chemotherapy of mCRC patients (Benson et al., 2017). However, patients could develop drug resistance to FOLFOX chemotherapy and then be exposed to chemotherapy-associated toxicities without any benefit. Therefore, a better understanding of the mechanism underlying resistance to FOLFOX chemotherapy would be helpful for the prevention and treatment of mCRC patients. In the era of individualized treatment, identifying valid predictive biomarkers chemotherapy resistance in mCRC is imperative.

Long non-coding RNAs (lncRNAs) play crucial roles in biological processes by regulating transcriptional modulation, splicing regulation, and posttranscriptional process (Fatica and Bozzoni, 2014; Anderson et al., 2015; Nelson et al., 2016). Accumulating evidence has also revealed that lncRNAs are implicated in the process of proliferation, invasion, progression, and metastasis of various cancers, including CRC (Fernández-Barrena et al., 2017; Li et al., 2017a; Dai et al., 2018; Shi et al., 2019). Recently, the potential function of lncRNAs as diagnostic and prognostic biomarkers of cancers has attached more and more attention from investigators (Fu et al., 2006; Sánchez and Huarte, 2013; Casero et al., 2015; Kurian et al., 2015; Jiang et al., 2017; Ali et al., 2018). However, studies are limited regarding lncRNAs associated with resistance to FOLFOX chemotherapy. Only a few lncRNAs were identified as effective biomarkers to FOLFOX chemotherapy resistance in mCRC (Li et al., 2017b, 2019).

Herein, lncRNA expression profiling was performed in mCRC patients receiving FOLFOX chemotherapy. Weighted gene coexpression network analysis (WGCNA) was then used to screen relevant hub lncRNA genes associated with FOLFOX chemoresistance. Finally, verification of hub genes was performed in other testing data (patient tissue samples).

## Materials and Methods

### Subjects

**Figure 1:**
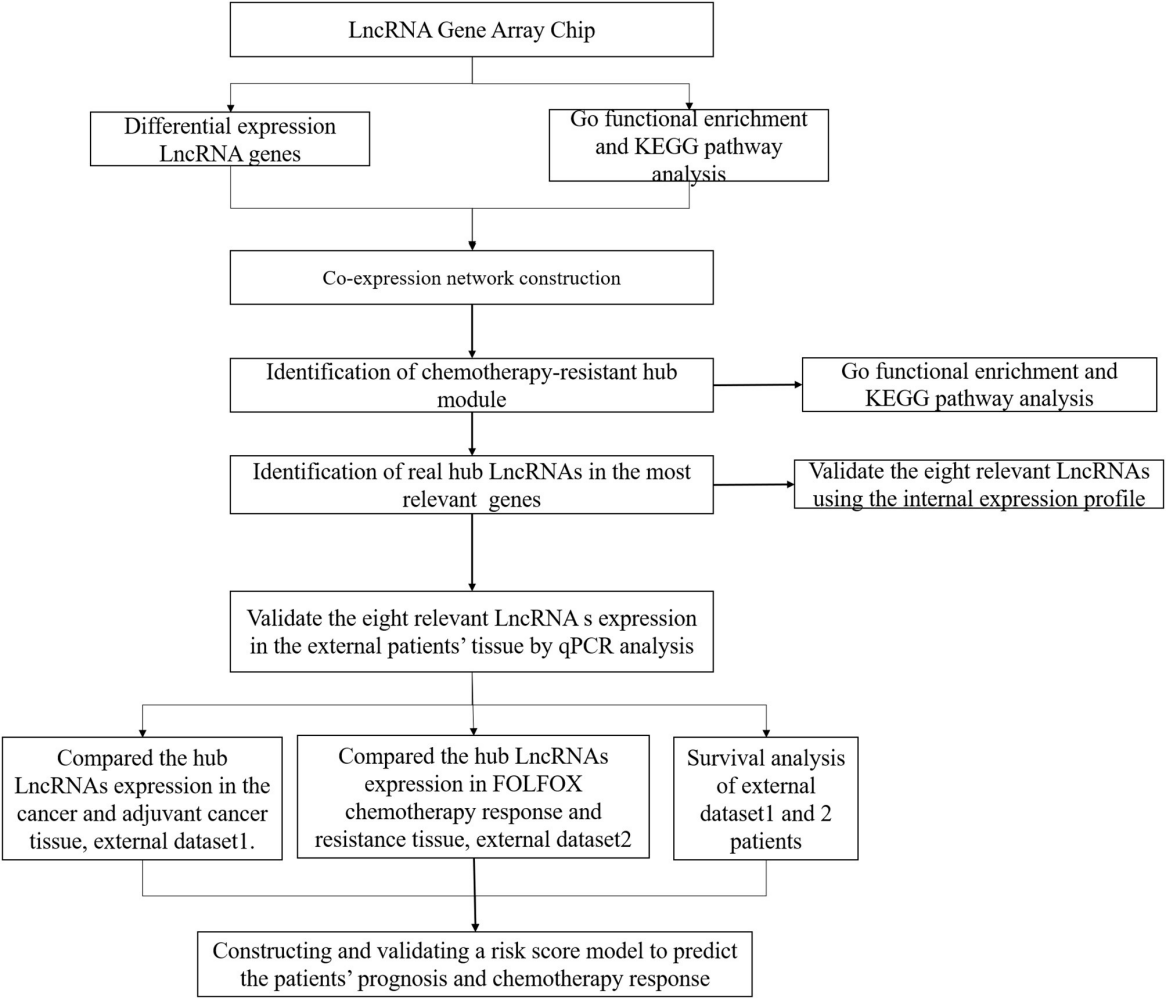
Workflow diagram in this study.

Between January 2017 and December 2017, 11 mCRC patients with synchronous liver metastases who received preoperative FOLFOX6 chemotherapy were enrolled in our study for lncRNA expression profiling (https://www.ncbi.nlm.nih.gov/geo/query/acc.cgi?acc=GSE138912, GSE138912), and the samples were collected at diagnosis by colonoscopy. After completion of six cycles of chemotherapy, the response to FOLFOX6 chemotherapy was evaluated using the Response Evaluation Criteria in Solid Tumors (RECIST) (Des Guetz et al., 2009; Ren et al., 2009). Briefly, the patients underwent CT/MR before and after FOLFOX6 chemotherapy to evaluate the size of the metastatic lesion, and tumor response was evaluated according to the cumulative length diameter value. Complete response (CR) means that all the metastatic lesions disappeared; partial response (PR) means that there is cumulative diameter reduction of more than 30% relative to a baseline value; disease progression (PD) means that cumulative diameter increase is >20% relative to baseline value or new metastatic lesion was found; and stable disease (SD) means that the cumulative length diameter of the metastatic lesion varies between PD and CR. Among them, four patients were included in the chemotherapy-sensitive group (CR, *n* = 0; PR, *n* = 4), while seven patients were included in the chemotherapy-resistant group (SD, *n* = 4; PD, *n* = 3). Moreover, a total of 136 without metastatic CRC patients in 2017 were used for building the risk score model and validating the lncRNAs expression in cancerous and adjacent cancerous tissues, named as the risk score training dataset, and the samples were collected after surgery. A total of 73 mCRC patients who received preoperative FOLFOX6 chemotherapy from 2017 to 2018 were included for external validation of predictive efficiency, named as the external validation dataset, and the samples were collected at diagnosis by colonoscopy. All the above samples were stored in liquid nitrogen for the further experiment. The study workflow is shown in Figure 1. Patient follow-up lasted until death or the cut-off date of September 30, 2019.

### RNA Extraction, Quality Control, Labeling, Array Hybridization, and Data Analysis

Total RNA extraction, quality control, labeling, and array hybridization were carried out according to our previous study (Zhang et al., 2020). The microarray was analyzed by Aksomics Inc. (Shanghai, China). Briefly, Agilent Feature Extraction software (version 10.7.3.1) and GeneSpring GX v11.5.1 software package (Agilent Technologies) were used for quantile normalization and subsequent data processing. Agilent Gene Spring GX software (version 11.5.1) was used for hierarchical clustering. The standard enrichment computation method was used for the Gene Ontology (GO) functional analysis and Kyoto Encyclopedia of Genes and Genomes (KEGG) pathway analysis.

### Co-expression Network Construction and Identification of Chemotherapy Sensitivity

The WGCNA algorithm was described in detail previously (Zhang and Horvath, 2005). Briefly, we first identified the qualification profiles for our data. We constructed the coexpression network by using the “WGCNA” package in R software (Horvath and Dong, 2008; Mason et al., 2009). Then, we established the correlation matrix and determined the soft threshold power by analyzing the network topology. Finally, the topological overlap matrix (TOM) was established (Yip and Horvath, 2007; Botía et al., 2017). Based on the phenotypic data of the groups, we calculated each module's *p*-value using a *t*-test gene significance.

To explore the relevant module, Pearson's correlation analysis was used to examine the association between module eigengenes (MEs) and chemotherapy resistance. To identify hub genes, we first chose the module with the highest correlation coefficient with the chemotherapy resistance (*P* < 0.05), and the genes that had the maximum absolute value of the Pearson's correlation in the module were defined as the hub genes.

### Gene Set Enrichment Analysis and Real-Time Quantitative Polymerase Chain Reaction

To figure out the potential function of the eight lncRNAs in mCRC patients, gene set enrichment analysis (GSEA) was performed in patients from our datasets. *P* < 0.05 and |enrichment score (ES)| > 0.3 were set as the cutoff criteria.

Total RNA extraction from patient tissues was according to the manufacturer's instruction (Invitrogen). One microgram total RNA was used for reverse transcription reaction using M-MLV Reverse Transcriptase Product (Promega). Real-time quantitative polymerase chain reaction (RT-qPCR) was performed using an ABI 7500 real-time PCR system (Thermo Fisher Scientific), and ASHGV40002660, ASHGV40041402, ASHGV40037204, ASHGV40000862, ASHGV40033167, ASHGV40021176, ASHGV40033762, and ASHGV40052035, lncRNA levels were assessed by RT-qPCR with glyceraldehyde 3-phosphate dehydrogenase (GAPDH) used as an internal control. PCR amplification was performed by denaturation at 94°C for 5 s, annealing, and extension at 62°C for 40 s for 40 cycles. The relative expression level of lncRNAs was calculated using the ΔCt method. In brief, the difference value between GAPDH Ct value and lncRNA Ct value was defined as the ΔCt value, and the high ΔCt value was recognized as the relatively low expression of the lncRNA in each sample. All PCR amplifications were performed in triplicate and repeated in three independent experiments. The RT-qPCR analysis was performed using primers in Supplementary Table 1.

### Internal and External Validation for the Hub lncRNAs

We first verified the hub lncRNAs expression in the chemotherapy-resistant and chemotherapy-sensitive groups in our data. Then, we further evaluated the hub lncRNAs expression between CRC and normal tissues and chemotherapy-resistant and chemotherapy-sensitive groups by using the external validating data. The receiver operating characteristic (ROC) curve was plotted, and the area under the ROC curve (AUC) was calculated to evaluate the predictive ability of the hub genes.

### Statistical Analyses

All statistical analyses were performed using SPSS software (version 23 SPSS Inc., Chicago, IL) and R software (version 3.4.1). The optimal cutoff values for lncRNAs expression were determined by using the X-tile program (Camp et al., 2004). Survival outcomes were assessed using the Kaplan–Meier method and the log-rank test. A Cox proportional hazards model was performed to identify risk factors for disease-free survival (DFS) (Friedman et al., 2010). Briefly, we calculated each sample risk score by using a risk score system. The patients were evenly divided into high- and low-risk groups based on the risk score. The performance of the model was evaluated by time-dependent ROC analysis, Kaplan–Meier curves, and Cox regression analysis. *P* < 0.05 was considered statistically significant.

## Results

### Cluster Analysis

**Figure 2:**
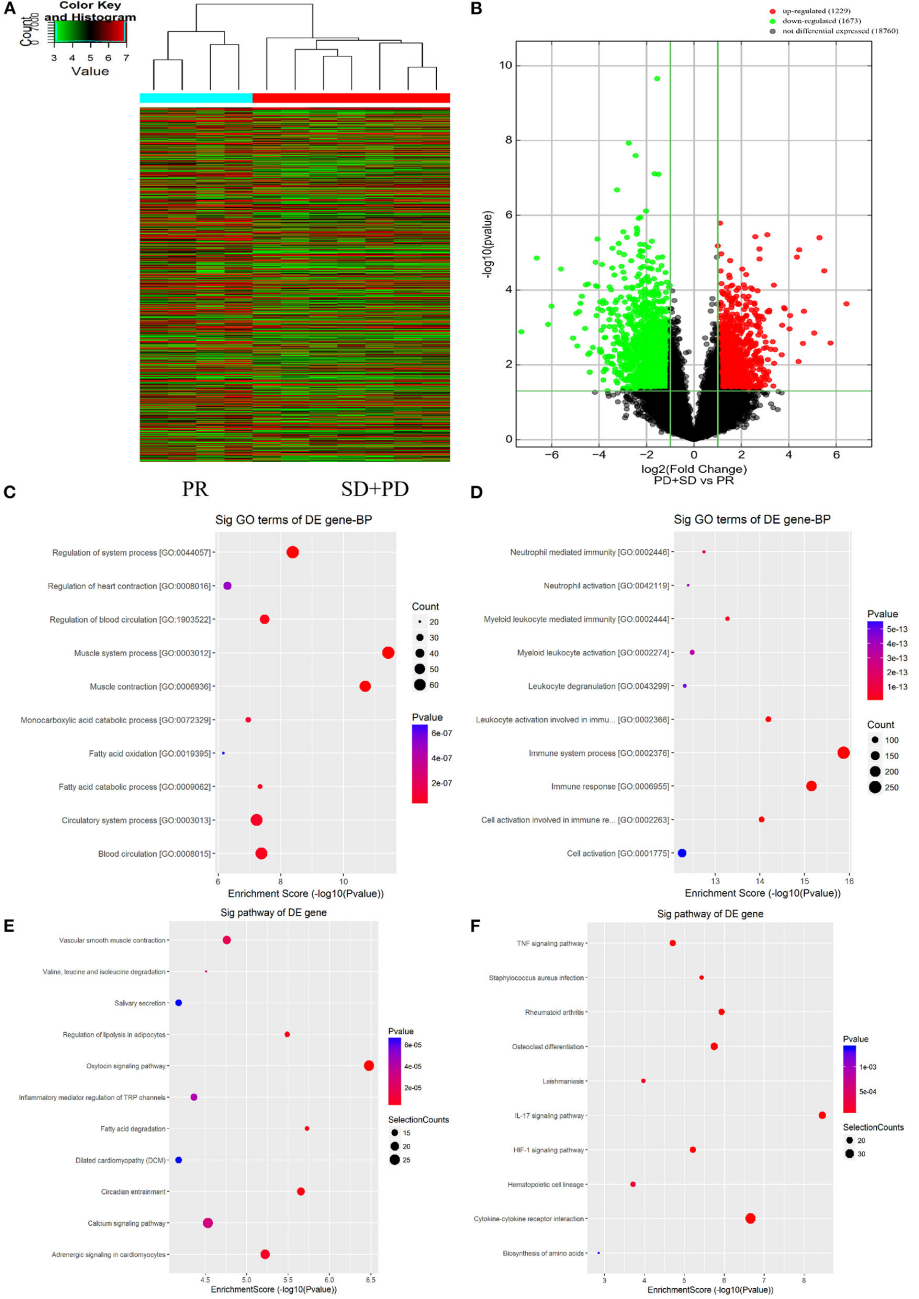
Long non-coding RNAs (lncRNAs) expression profile comparison between chemotherapy-resistant and chemotherapy-sensitive groups. Gene Ontology (GO) functional and Kyoto Encyclopedia of Genes and Genomes (KEGG) pathway analysis of the differentially expressed genes. **(A)** The hierarchical clustering of all target values of lncRNA expression profiling among samples. **(B)** Between the chemotherapy-resistance and chemotherapy-sensitivity group. The purple dots indicated the upregulated genes of messenger RNAs (mRNAs), and the green dots indicated the downregulated genes of mRNAs. **(C)** GO functional analysis of the top 10 functional classifications of the upregulated genes. **(D)** GO functional analysis of the top 10 functional classifications of the downregulated genes. **(E)** KEGG pathway analysis of the top 10 significant pathways of upregulated genes. **(F)** KEGG pathway analysis of the top 10 pathways of downregulated genes.

Gene expression profiling in primary tumor cells was performed using the Agilent lncRNA Gene Chip Array. A total of 45,580 lncRNAs were detected. Supervised hierarchical cluster analysis demonstrated a clustering trend between the two groups (Figures 2A,B). The sample for differentially expressed genes (DEGs) demonstrated that tumor cell biology significantly differed between the two groups, chemotherapy-resistant group vs. chemotherapy-sensitive group, including 24 upregulated and 89 downregulated genes [all false discovery rate (FDR) < 0.01].

### GO Enrichment and KEGG Pathway Analysis

The molecular mechanism of differentially expressed lncRNAs (DElncRNAs) involved in FOLFOX chemoresistance for mCRC patients was studied by using GO enrichment analysis. We evaluated the top 10 significant GO terms enriched in DEGs in mCRC patients (Figures 2C,D). The top three significant GO terms in the upregulated genes were related to the system process, heart contraction, and regulation of blood circulation, whereas the top three significant GO terms in the downregulated genes were related to neutrophil-mediated immunity, neutrophil activation, and myeloid leukocyte mediated immunity.

As shown in Figures 2E,F, KEGG analysis demonstrated that the top three upregulated genes were associated with the vascular smooth muscle contraction, valine, leucine, and isoleucine degradation, and salivary secretion signaling pathway. The top three downregulated genes were related to the tumor necrosis factor (TNF) signaling pathway, *Staphylococcus aureus* infection, and rheumatoid arthritis signaling pathway.

### WGCNA

**Figure 3:**
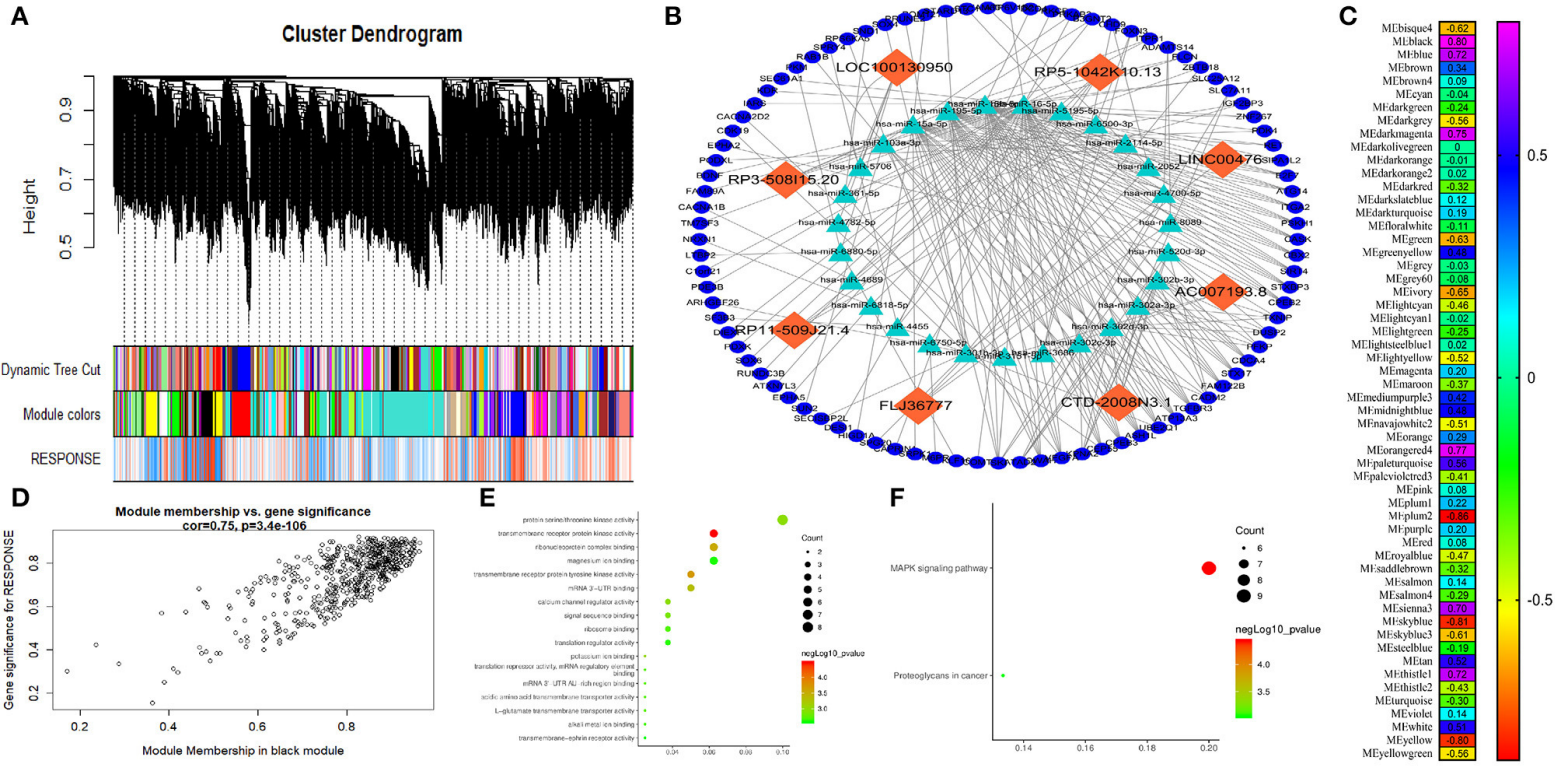
Weighted correlation network analysis (WGCNA) and hub gene screened. **(A)** Dendrogram of all expressed genes in the top 25% of variance clustered based on a dissimilarity measure (1 – TOM). **(B)** The mechanism of the hub long non-coding RNAs (lncRNAs). **(C)** Heatmap of the correlation between module eigengenes and chemotherapy resistance. **(D)** Scatter plot of the correlation between the black module and chemotherapy resistance. **(E)** Gee Ontology (GO) functional analysis of the top 10 pathways of genes in the black modules. **(F)** Kyoto Encyclopedia of Genes and Genomes (KEGG) pathway analysis of the top 10 pathways of genes in the black modules.

A weighted coexpression network was built to further identify the hub genes (Figure 3A), and 56 modules were identified, as shown in Figure 3C. We also analyzed the relationship between chemoresistance and modules. Among these modules, the module eigengene (ME) of the black module had the highest positive correlation with chemoresistance (*r* = 0.80, *P* < 0.001), while the ME of the plum2 module had the highest negative correlation with chemoresistance (*r* = −0.86, *P* < 0.001). Through WGCNA, 582 genes in the black module were identified as genes with high module connectivity. Then, Pearson's test was used to further explore the association between each gene and chemoresistance (Figure 3D). The most eight relevant lncRNAs (ASHGV40002660, AC007193.8; ASHGV40041402, CTD-2008N3.1; ASHGV40037204, FLJ36777; ASHGV40000862, RP11-509J21.4; ASHGV40033167, RP3-508I15.20; ASHGV40021176, LOC100130950; ASHGV40033762, RP5-1042K10.13; and ASHGV40052035, LINC00476) were selected as the hub lncRNAs. To further evaluate the function of eight lncRNAs, we analyzed a previous dataset (GSE138912) and constructed a ceRNA network (Figure 3B). GO analysis was performed to evaluate the potential biological functions of the lncRNAs (Figure 3E). Additionally, we evaluated the most eight relevant lncRNAs by KEGG analysis. The pathways were related to the proteoglycans in cancer and the MAPK signaling pathway (Figure 3F). Moreover, GSEA was conducted to determine the potential mechanism for the eight lncRNAs involvement in chemotherapy resistance in CRC. Our data demonstrated that the enriched correlated KEGG pathways included the small cell lung cancer, calcium signal pathway, and propanoate metabolism, as shown in Supplementary Figure 2.

### Hub LncRNAs Identification and Validation in the Internal Expression Profile

**Figure 4:**
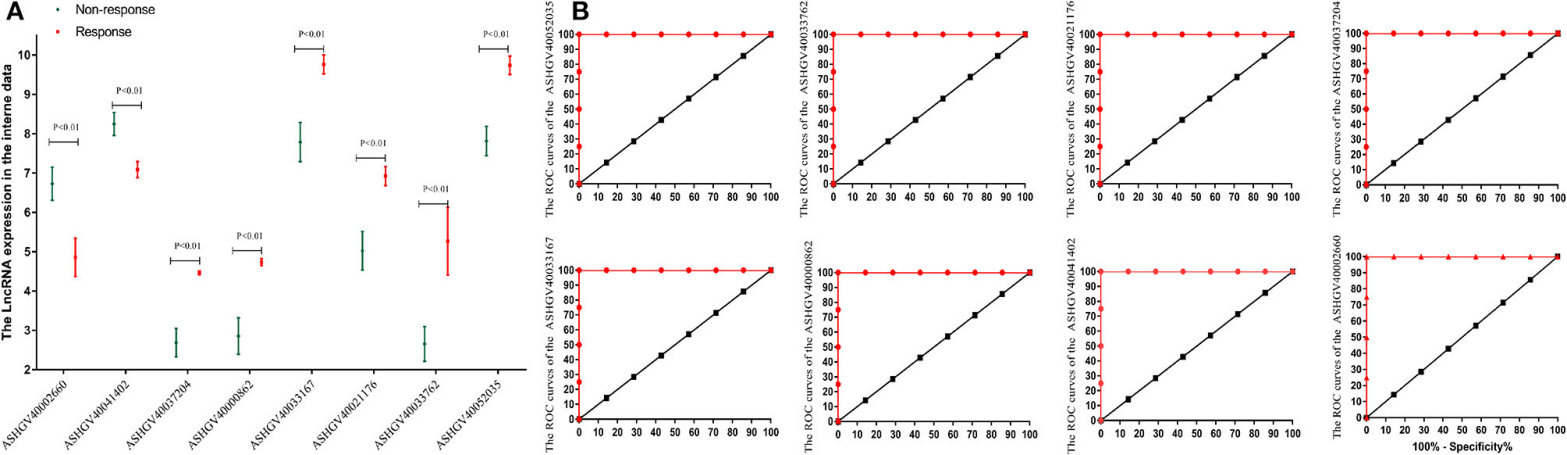
Internal validation of hub lncRNAs. **(A)** In our data the hub long non-coding RNAs (lncRNAs) expression (all *P* < 0.01). **(B)** Receiver operating characteristic (ROC) analysis to evaluate the predictive efficiency of the hub lncRNAs using our data set.

To further identify the hub genes, we analyzed the expression of hub genes between the two groups. The results in Figure 4A demonstrated that the expression of hub genes ASHGV40002660 and ASHGV40041402 was higher in the chemotherapy-resistant group (6.73 ± 0.42 vs. 4.86 ± 0.49, *P* < 0.001; 8.25 ± 0.29 vs. 7.09 ± 0.21, *P* < 0.001). The expression of ASHGV40037204, ASHGV40000862, ASHGV40033167, ASHGV40021176, ASHGV40033762, and ASHGV40052035 was lower in the chemotherapy-resistant group (2.69 ± 0.36 vs. 4.47 ± 0.05, *P* < 0.001; 2.86 ± 0.46 vs. 4.74 ± 0.09, *P* < 0.001; 7.79 ± 0.50 vs. 9.76 ± 0.24, *P* < 0.001; 5.03 ± 0.49 vs. 6.92 ± 0.24, *P* < 0.001; 2.66 ± 0.44 vs. 5.27 ± 0.86, *P* < 0.001; 7.82 ± 0.37 vs. 9.74 ± 0.23, *P* < 0.001). ROC analysis demonstrated that all hub genes had a predictive ability in predicting chemoresistance to FOLFOX chemotherapy for mCRC patients (all *P* < 0.001, AUC = 1, Figure 4B).

### Hub LncRNAs Validation in the Non-metastatic CRC Dataset and Dataset Cutoff Values for Hub lncRNAs

**Figure 5:**
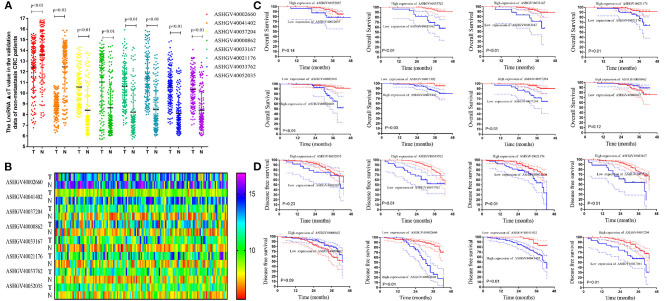
External validation of hub long non-coding RNAs (lncRNAs) in the non-metastatic colorectal cancer (CRC) patients. **(A)** The hub lncRNAs ΔCt value in the cancerous tissue and adjacent non-cancer tissue in external CRC patients by quantitative PCR (qPCR) (all *P* < 0.01). **(B)** The heatmap of the ΔCt value. **(C)** The Kaplan–Meier analysis for the overall survival of the hub lncRNAs in the non-metastatic CRC patients. **(D)** The Kaplan–Meier analysis for the disease-free survival of the hub lncRNAs in the non-metastatic CRC patients.

To independently validate the hub genes, we analyzed the expression level of the lncRNAs between the cancerous and adjacent non-cancerous tissues using qPCR (Figures 5A,B). A total of the 136 non-metastatic CRC patients were enrolled in the present study as the external validation dataset, named as the external dataset 1. The clinicopathological characteristics of patients are summarized in Supplementary Table 2. The results demonstrated that the expression of ASHGV40002660 and ASHGV40041402 were lower in the cancerous tissues (12.44 ± 2.37 vs. 13.55 ± 2.38, *P* < 0.001; 8.78 ± 1.02 vs. 12.46 ± 2.35, *P* < 0.001). The expression of ASHGV40037204, ASHGV40000862, ASHGV40033167, ASHGV40021176, ASHGV40033762, and ASHGV40052035 was higher in the cancerous tissues than in the adjacent non-cancerous tissues (10.58 ± 1.80 vs. 8.04 ± 2.28, *P* < 0.001; 11.04 ± 2.70 vs. 8.63 ± 2.30, *P* < 0.001; 10.64 ± 1.98 vs. 8.22 ± 2.10, *P* < 0.001; 11.41 ± 2.15 vs. 8.47 ± 2.09, *P* < 0.001; 10.55 ± 1.77 vs. 8.12 ± 1.68, *P* < 0.001; 10.36 ± 1.63 vs. 8.41 ± 1.79, *P* < 0.001).

**Figure 6:**
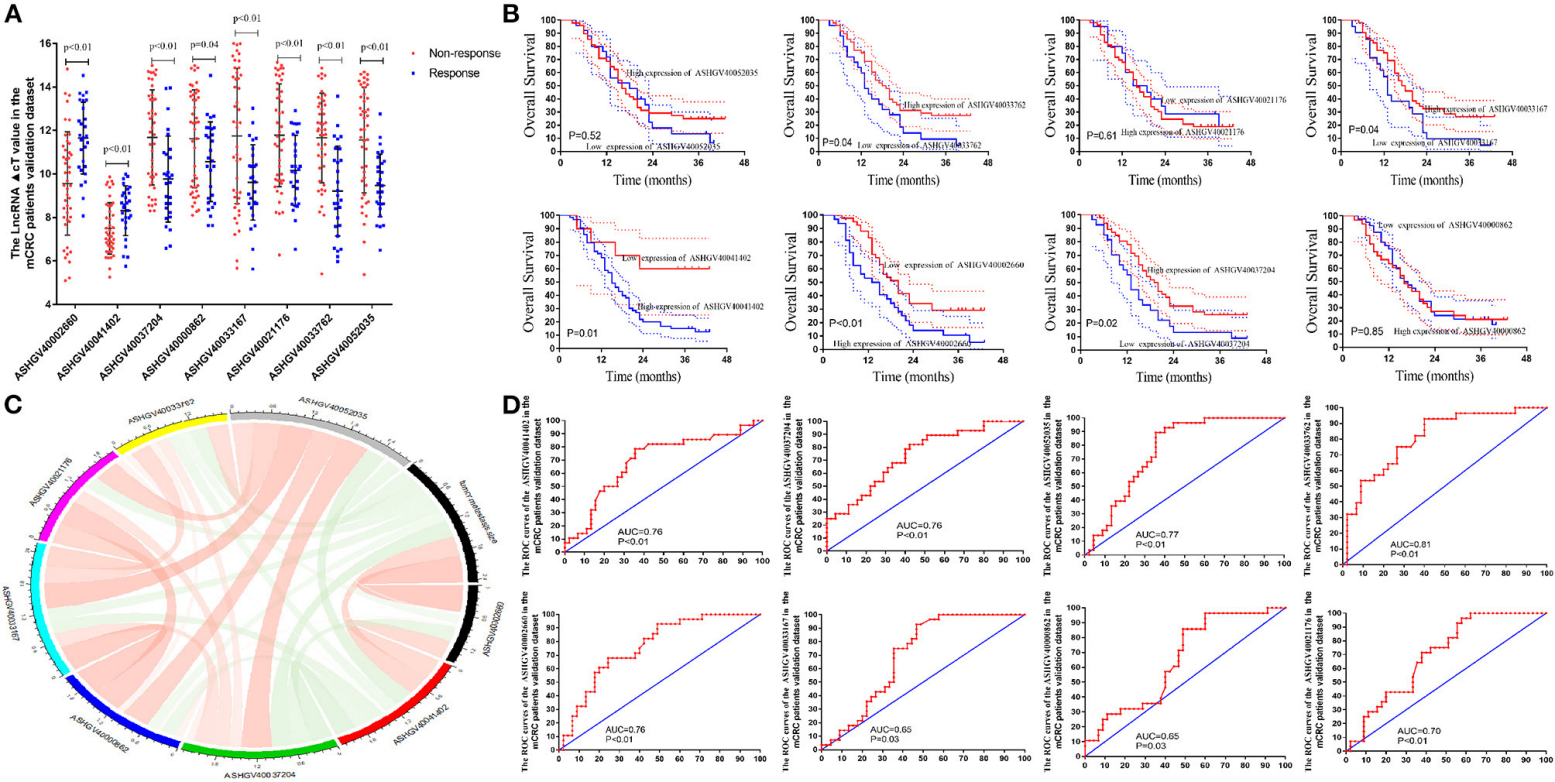
External validation of hub long non-coding RNAs (lncRNAs) in metastatic colorectal cancer (mCRC) patients. **(A)** The hub lncRNAs ΔCt value in the chemotherapy-sensitive and chemotherapy-resistance tissue in external mCRC patients by quantitative PCR (qPCR) (all *P* < 0.01). **(B)** Correlation analysis between the hub lncRNAs expression and tumor response to FOLFOX chemotherapy. **(C)** The Kaplan–Meier analysis for the overall survival of the hub lncRNAs in mCRC patients. **(D)** Receiver operating characteristic (ROC) analysis to evaluate the predictive efficiency of the hub LncRNAs in mCRC patients for FOLFOX chemotherapy.

The X-tile analysis was used to determine the optimal cutoff values in terms of DFS. As seen in Figure 6A and Supplementary Figure 1, X-tile plots identified 11.0, 10.3, 12.7, 12.9, 12.0, 11.9, 9.4, and 12.0 as cutoff values for ASHGV40000862, ASHGV40002660, ASHGV40021176, ASHGV40033167, ASHGV40033762, ASHGV40037204, ASHGV40041402, and ASHGV40052035, respectively. Accordingly, the entire cohort was divided into low and high subgroups.

Lower expression of ASHGV40002660 and ASHGV40041402 correlated with a better prognosis in CRC patients (*P* < 0.01 and *P* = 0.03, Figure 5C). Noticeably, a higher expression of ASHGV40037204, ASHGV40033167, ASHGV40021176, and ASHGV40033762 was correlated with an improved OS (both *P* < 0.01), as shown in Figure 5C. The OS rates were similar in the high and low ASHGV40052035 and ASHGV40000862 expression groups (*P* = 0.14 and *P* = 0.12).

Lower expression of ASHGV40002660 and ASHGV40041402 correlated with a better prognosis in CRC patients (both *P* < 0.01, Figure 5D). Noticeably, a higher expression of ASHGV40037204, ASHGV40033167, ASHGV40021176, and ASHGV40033762 was correlated with an improved DFS (both *P* < 0.01), as depicted in Figure 5C. The OS rates were similar in the high and low ASHGV40052035 and ASHGV40000862 expression groups (*P* = 0.23 and *P* = 0.09). Moreover, multivariate Cox regression analysis was performed to explore the independent predictive factors of the eight lncRNAs. The results demonstrated that ASHGV40002660 [hazard ratio (HR) = 0.681, 95%CI 0.593–0.782, *P* < 0.001], ASHGV40041402 (HR = 0.655, 95%CI 0.451–0.949, *P* = 0.025), and ASHGV40033762 (HR = 1.241, 95%CI 1.009–1.525, *P* = 0.041) were independent predictors of CRC patients' DFS, as shown in Table 1. We found a similar results of the multivariate Cox regression analysis in OS. The results demonstrated that ASHGV40002660 (HR = 0.709, 95%CI 0.564–0.892, *P* = 0.003) and ASHGV40033762 (HR = 1.692, 95%CI 1.181–2.424, *P* = 0.004) were independent predictors of CRC patients' OS, as shown in Table 1.

**Table 1:** Cox regression analysis of eight long non-coding RNAs (lncRNAs) for disease-free survival and overall survival in colorectal cancer (CRC) patients (*n* = 136).

	**Disease free survival**			**Overall survival**		
**Variables**	**Multivariate analysis**			**Multivariate analysis**		
	**HR**	**95% CI**	***P*-value**	**HR**	**95% CI**	***P*-value**
ASHGV40002660	0.681	0.593−0.782	<0.001	0.709	0.564−0.8911	0.003
ASHGV40041402	0.655	0.451−0.949	0.025	0.656	0.347−1.241	0.195
ASHGV40037204	1.11	0.915−1.346	0.289	0.97	0.701−1.343	0.855
ASHGV40000862	0.978	0.873−1.095	0.698	0.897	0.732−1.099	0.293
ASHGV40033167	1.066	0.91−1.248	0.431	0.882	0.673−1.157	0.364
ASHGV40021176	1.028	0.883−1.196	0.725	0.942	0.7−1.268	0.696
ASHGV40033762	1.241	1.009−1.525	0.041	1.692	1.181−2.424	0.004
ASHGV40052035	0.968	0.784−1.196	0.764	1.277	0.884−1.845	0.192

### Hub lncRNAs Validation in the mCRC External Dataset

To independently validate the predictive efficiency of the hub genes, we analyzed the lncRNAs expression levels in the cancerous tissues in mCRC patients treated with FOLFOX neo-chemotherapy using qPCR (Figure 6A). A total of the 73 mCRC patients (48 male and 25 female) were enrolled in the present study as the external validation dataset, named as the external dataset 2. The patients' clinical and pathological features are listed in Supplementary Table 2. Among them, 25 patients were included in the chemotherapy-sensitive group (CR, *n* = 0; PR, *n* = 25), while 48 patients were included in the chemotherapy-resistant group (SD, *n* = 20; PD, *n* = 28). Based on the RECIST criterion, we analyzed the relationship between hub lncRNA expression and the tumor response to chemotherapy (Figure 6B). The results demonstrated that the expression value of the ASHGV40002660, ASHGV40033167, ASHGV40033762, ASHGV40037204, ASHGV40041402, and ASHGV40052035 were associated with the tumor response (*r* = 0.37, *P* < 0.001; *r* = −0.186, *P* = 0.021; *r* = −0.257, *P* = 0.001; *r* = −0.239, *P* < 0.001; *r* = 0.285, *P* < 0.001; *r* = −0.208, *P* = 0.010). ASHGV40000862 and ASHGV40021176 had no significant association with tumor response (*r* = −0.155, *P* = 0.053; *r* = −0.157, *P* = 0.051). Additionally, we analyzed the hub lncRNAs expression in the chemotherapy-sensitive and chemotherapy-resistance groups (Figure 6A). The results demonstrated that the expression of ASHGV40002660 and ASHGV40041402 were higher in the chemotherapy-resistant tissues than in the chemotherapy-sensitive tissues (9.56 ± 2.38 vs. 11.65 ± 1.65, *P* < 0.001; 7.50 ± 1.18 vs. 8.31 ± 1.14, *P* = 0.005). The expression of ASHGV40037204, ASHGV40000862, ASHGV40033167, ASHGV40021176, ASHGV40033762, and ASHGV40052035 was lower in the chemotherapy-resistant tissues than in the chemotherapy-sensitive tissues (11.69 ± 2.17 vs. 9.77 ± 1.97, *P* < 0.001; 11.62 ± 2.25 vs. 10.57 ± 1.83, *P* = 0.040; 11.75 ± 3.11 vs. 9.61 ± 1.73, *P* = 0.001; 11.78 ± 2.36 vs. 10.17 ± 1.61, *P* = 0.002; 11.66 ± 2.06 vs. 9.22 ± 2.06, *P* = 0.001; 11.56 ± 2.43 vs. 9.47 ± 1.44, *P* < 0.001).

Based on the above-mentioned X-tile analysis results, we divided the lncRNAs into low- and high-expression groups, and Kaplan–Meier analysis was performed to analyze the prognosis of mCRC patients. The results revealed that the lower expression of ASHGV40002660 and ASHGV40041402 were associated with a better prognosis in mCRC patients (*P* < 0.01 and *P* = 0.03, Figure 6C). Noticeably, higher expression of ASHGV40037204, ASHGV40033167, and ASHGV40033762, was correlated with an improved OS (*P* = 0.02, *P* = 0.04, and *P* = 0.04), as shown in Figure 6C. The OS rates were similar in the high and low ASHGV40021176, ASHGV40052035, and ASHGV40000862 expression groups (*P* = 0.61, *P* = 0.52, and *P* = 0.85).

Moreover, the predictive ability of each hub lncRNA in patients receiving FOLFOX chemotherapy before surgery was further explored. The hub gene with the biggest predictive power was ASHGV40033762 (AUC = 0.81, *P* < 0.01, Figure 6D). The predictive ability of other lncRNAs, such as ASHGV40002660 (AUC = 0.76, *P* < 0.01), ASHGV40037204 (AUC = 0.76, *P* < 0.01), ASHGV40000862 (AUC = 0.65, *P* = 0.03), ASHGV40033167 (AUC = 0.65, *P* = 0.03), ASHGV40021176 (AUC = 0.70, *P* < 0.01), ASHGV40041402 (AUC = 0.76, *P* < 0.01), and ASHGV40052035 (AUC = 0.77, *P* < 0.01) were also described in Figure 6D.

### Construction of a Risk Factor Model

**Figure 7:**
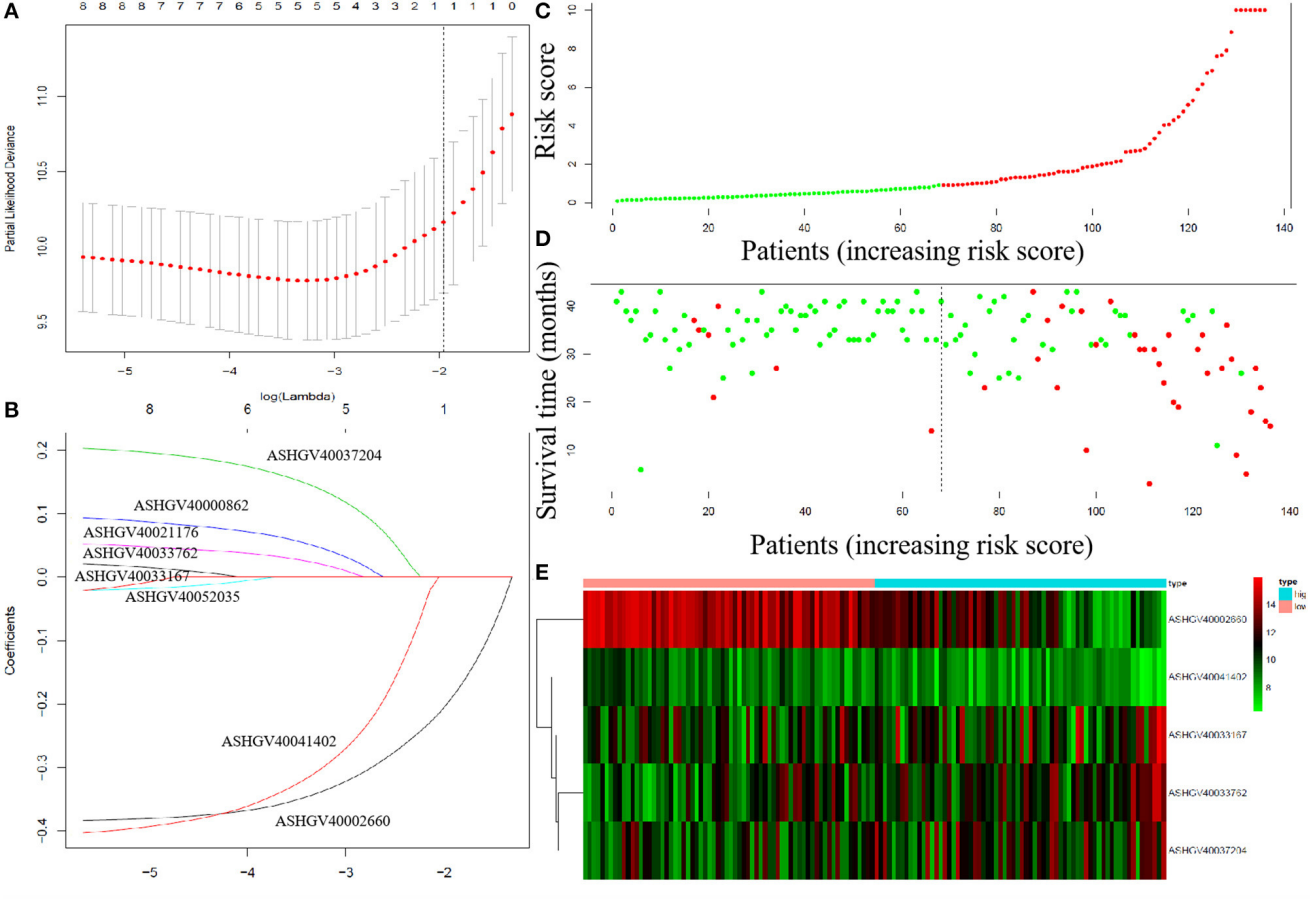
The least absolute shrinkage and selection operator (LASSO) analysis and risk score system were constructed. **(A)** The area under the ROC curve (AUC) was estimated with a cross-validation technique, and the largest lambda value was chosen when the cross-validation error was within one standard error of the minimum. **(B)** LASSO coefficient profiles of the eight factors. **(C–E)** The risk factor model of the hub lncRNAs in the non-metastatic CRC patients. **(C)** Long non-coding RNAs (lncRNAs) risk score distribution of 136 CRC patients. **(D)** Survival status in non-metastatic CRC patients (*N* = 136). **(E)** Heatmap of the hub lncRNAs expression. Red: high expression; blue: low expression.

To explore the prognostic impact of the hub lncRNAs on DFS in non-metastatic CRC patients, we performed Cox regression analysis and least absolute shrinkage and selection operator (LASSO) analysis to explore the significant risk factors for DFS. The results revealed that ASHGV40002660, ASHGV40033167, ASHGV40033762, ASHGV40037204, and ASHGV40041402 were significant factors (Figures 7A,B). Based on the significant predictors in the LASSO analysis, the risk score model for DFS in mCRC patients was developed, as demonstrated in Figures 7C–E. The hub lncRNAs risk score system was constructed using the formula as follows: risk score = (−0.40) × (ΔCt value of ASHGV40002660) + [−0.41 × (ΔCt value of ASHGV40041402) + 0.10 × (ΔCt value of ASHGV40037204) + 0.05 × (ΔCt value of ASHGV40033167) + 0.21 × (ΔCt value of ASHGV40033762)]. Accordingly, each patient had a risk score that was associated with an individual prognosis. The cutoff value was determined as 0.91 for risk scores by using ROC analysis; thus, the patients were separated into high- and low-risk groups (Figures 7B–D). Based on the risk group and patients' prognosis, we drew the survival plot (Figure 7D). Additionally, the lncRNAs expression data were displayed in the order of the risk score in Figure 7E.

### Prognostic Value of the Risk Score and a Nomogram Model Was Constructed in the Non-metastatic CRC Patients and Validation of the Risk Score in the External Datasets

**Figure 8:**
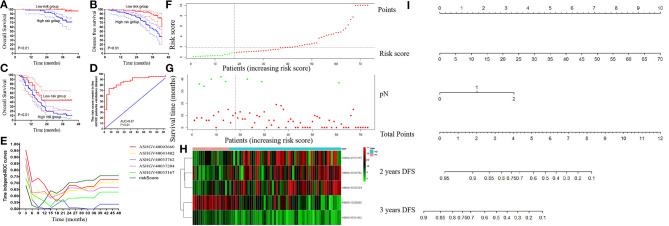
External validation of risk score in the non-metastatic colorectal cancer (CRC) and metastatic colorectal cancer (mCRC) patients. **(A,B)** The Kaplan–Meier analysis for the overall survival **(A)** and disease-free survival **(B)** of the risk score in the non-metastatic CRC patients. **(C)** The Kaplan–Meier analysis for the overall survival of the risk score in mCRC patients. **(D)** Receiver operating characteristic (ROC) curves and area under the ROC curve (AUC) analysis to evaluate the predictive efficiency of the risk score in mCRC patients for FOLFOX chemotherapy. **(E)** Time-dependent AUC curves of the hub lncRNAs and risk factor models for the prediction of disease-free survival (DFS) in the non-metastatic CRC patients. **(F–H)** The risk factor model of the hub lncRNAs in mCRC patients. **(F)** LncRNA risk score distribution of 73 mCRC patients. **(G)** Survival status in mCRC patients (*N* = 73). **(H)** Heatmap of the hub lncRNAs expression. Red: high expression; blue: low expression. **(I)** Nomogram developed for prediction of disease-free survival in the non-metastatic CRC patients.

Cox regression analysis was performed to explore the prognostic impact of risk score on DFS in non-metastatic CRC patients. Univariate analysis showed that American Society of Anesthesiologists (ASA, *P* = 0.010), pathological T stage (*P* = 0.042), pathological *N* stage (*P* < 0.001), risk score (*P* < 0.001), perineural invasion (*P* = 0.042), and vascular invasion (*P* = 0.010) were independently associated with DFS in non-metastatic CRC (Table 2). COX analysis showed that pathological *N* stage (HR = 1.717, 95%CI 1.118–2.638, *P* = 0.013) and risk score (HR = 1.079, 95%CI 1.051–1.108, *P* < 0.001) were independent predictors of DFS following NCRT, as shown in Table 1. Then, a nomogram model was constructed to predict the prognosis of the non-metastatic CRC patients, as shown in Figure 8I.

**Table 2:** Cox regression analysis of predictive factors for disease-free survival in colorectal cancer (CRC) patients (*n* = 136).

**Variables**	**Univariate analysis**			**Multivariate analysis**		
	**HR**	**95% CI**	***P*-value**	**HR**	**95% CI**	***P*-value**
Sex, male/female	1.103	0.591–2.057	0.758			
Age	0.979	0.954–1.005	0.109			
ASA	0.467	0.261–0.835	0.010	0.586	0.337–1.021	0.059
Tumor size	0.946	0.818–1.095	0.460			
Pathological T stage	1.851	1.022–3.351	0.042	1.423	0.737–2.745	0.293
Pathological N stage	2.360	1.629–3.418	<0.001	1.717	1.118–2.638	0.013
BMI	1.011	0.923–1.108	0.807			
Postoperative hospital stay	0.997	0.950–1.047	0.908			
Tumor location			0.964			
Ascending colon	Reference	Reference				
Transverse colon	1.059	0.336–3.340	0.922			
Descending colon	1.141	0.354–3.680	0.826			
Sigmoid colon	0.954	0.351–2.544	0.910			
Rectum	0.810	0.326–2.014	0.650			
CEA level	1.458	0.781–2.722	0.237			
CA19-9 level	1.657	0.724–3.794	0.232			
Risk score	1.233	1.167–1.302	<0.001	1.079	1.051–1.108	<0.001
Nerval invasion	1.973	1.027–3.791	0.042	1.665	0.828–3.348	0.153
Vascular invasion	3.266	1.330–8.020	0.010	2.025	0.766–5.354	0.153
Tumor differentiation	1.429	0.902–2.264	0.128			

*CRC, colorectal cancer; HR, hazard ratio; CI, confidential interval; ASA, American Society of Anesthesiologists; BMI, body mass index*.

Using the risk score formula, we calculated each mCRC patients' risk score, and the mCRC patients were divided into the low- and high-risk groups based on the cutoff value of 0.91 (Figures 8F–H). Moreover, Kaplan–Meier analysis was carried out to compare the prognosis of patients in the low- and high-risk groups in both non-metastatic CRC patients' dataset and mCRC patients' dataset. In the non-metastatic CRC patients' dataset, the 3-years OS and DFS rates were significantly higher in the low-risk score group than in the high-risk score group (100 vs. 75.25%, 89.59 vs.55.62%, respectively, both *P* < 0.01; Figures 8A,B). Notably, in the mCRC patients' dataset, the 3-years OS rates in the low-risk score group were 44.44%, significantly higher than 13.52% in the high-risk score group (*P* = 0.01), as shown in Figure 8C. The ROC curve revealed that the risk score system had powerful predictive ability in predicting the FOLFOX chemotherapy response in mCRC patients (AUC = 0.87, *P* < 0.01, Figure 8D).

Time-dependent AUC curves demonstrated that the AUCs of all the hub lncRNAs were relatively stable after surgery. As depicted in Figure 8E, ASHGV40002660 had the most powerful predictive ability among all the hub lncRNAs. Moreover, the risk score system showed a stronger predictive ability to predict OS for non-metastatic CRC patients than any single hub lncRNA.

## Discussion

FOLFOX chemoresistance is a tough problem in the treatment of CRC patients. Thus, identifying reliable diagnostic and prognostic biomarkers for FOLFOX chemoresistance becomes imperative. Through WGCNA, an advanced methodology of multigene analysis, the present study for the first time identified gene coexpression modules related to FOLFOX chemoresistance based on lncRNAs microarray. Eight hub lncRNAs were selected, including ASHGV40002660, ASHGV40041402, ASHGV40037204, ASHGV40000862, ASHGV40033167, ASHGV40021176, ASHGV40033762, and ASHGV40052035. The eight lncRNAs had a powerful ability to predict FOLFOX chemoresistance. Moreover, we employed 196 CRC patients' cancerous tissue and adjacent non-cancerous tissues as the external validation dataset. A lncRNA risk score model predicting FOLFOX chemoresistance and prognosis of CRC patients was constructed.

The role of lncRNA as potential powerful biomarker has been reported in several cancers, including CRC (Chi et al., 2019; Pichler et al., 2020; Rahmani et al., 2020; Wang et al., 2020; Wu et al., 2020). In previous studies, lncRNAs can act as the biomarker for diagnosis and prediction of the prognosis and progression in CRC patients (Alidoust et al., 2018; Wei et al., 2019; Pichler et al., 2020). However, the function and predictive effect of lncRNAs in FOLFOX chemotherapy resistance are still unclear. To explore the role of the lncRNAs in the mCRC patients receiving FOLFOX chemotherapy, we employed the lncRNAs microarray expression profiling, which detected over 45,000 reliable lncRNAs to detect the DElncRNAs in 11 mCRC patients. The results demonstrated that a total of the 113 DElncRNAs were identified between the FOLFOX regimen sensitive/resistant group (*P* < 0.05, fold change > 2). The function of the DElncRNAs was associated with the TNF signaling pathway and neutrophil-related immune regulation in GO and KEGG analyses.

Currently, WGCNA has emerged as an effective method to discover the relationship between networks/genes, phenotypes, and samples' biological information to avoid the defects of the traditional method (Gao et al., 2016; Bakhtiarizadeh et al., 2018; Magani et al., 2018). It can also be used to bridge gaps between individual genes and the occurrence and progression of diseases (Zhang and Horvath, 2005; Langfelder and Horvath, 2008; Tian et al., 2017). Additionally, WGCNA facilitates network-based gene screening methods, which can be used to identify and screen key biomarkers associated with clinical traits in various cancers (Citations). However, this efficient bioinformatics approach has not yet been adopted to identify network-centric lncRNA genes associated with FOLFOX chemotherapy-resistant mCRC. Thus, we performed WGCNA to identify the “real” hub gene. The results from the WGCNA revealed the eight most relevant lncRNAs and had a strong ability to predict the FOLFOX regimen sensitivity in the internal validation by ROC curve and expression value analysis. Moreover, to further explore the function of the eight hub lncRNAs, we combined them with our previous mRNA dataset (GSE138912), which originated from the same sample of patients, to analyze the underlying mechanism. Based on the mechanism of lncRNA–miRNA–mRNA/lncRNA–mRNA action (Kopp and Mendell, 2018; Krause, 2018), a total of 89 mRNAs were selected. Then, the hub lncRNAs function was evaluated by GO and KEGG analyses. The results demonstrated that the MAPK signaling pathway and protein biological regulation were the most relevant functional, which was consistent with previous studies (Belli et al., 2019; Schumacher et al., 2019; Vitiello et al., 2019).

To further verify the hub lncRNAs screened by the lncRNA microarray profiling and WGCNA, we examined the hub lncRNAs expression in the cancerous and adjacent non-cancerous tissues in the external 136 CRC patients. The results demonstrated a higher expression of ASHGV40002660 and ASHGV40041402 in the cancerous tissues. The expression of ASHGV40037204, ASHGV40000862, ASHGV40033167, ASHGV40021176, ASHGV40033762, and ASHGV40052035 were lower in the cancerous tissues. Moreover, high expression of ASHGV40002660 and ASHGV40041402 was associated with a shorter DFS. Contrarily, high expression of ASHGV40037204, ASHGV40021176, ASHGV40033762, and ASHGV40033167 indicated a longer DFS. Then, to improve the predictive ability of the hub lncRNAs in predicting CRC patients' prognosis, a risk factor model was constructed based on the proportion of each variable in the Cox regression model. With a risk score formula, patients were divided into high- and low-risk groups. The risk score predicting model has been proposed as a tool for prognosis prediction in several types of cancers, including colon cancers (Dai et al., 2018; Gu et al., 2018; Liao et al., 2018). However, no study has focused on the prognosis of non-metastatic and metastatic CRC patients. Herein, we built a risk score model based on a five-lncRNA signature that had a powerful ability in predicting the non-metastatic and metastatic CRC patient's survival. Moreover, the result was also verified in the time-dependent ROC analysis, indicating resistance to FOLFOX chemotherapy.

Additionally, to evaluate the association of the eight lncRNAs with FOLFOX chemotherapy in mCRC patients, we screened out 73 CRC patients who received the FOLFOX chemotherapy before surgery in an external data set. The results demonstrated that the ASHGV40002660 and ASHGV40041402 were higher in the FOLFOX chemotherapy-resistant cancerous tissues than in the sensitive cancerous tissues. The expression of ASHGV40037204, ASHGV40000862, ASHGV40033167, ASHGV40021176, ASHGV40033762, and ASHGV40052035 were lower in the FOLFOX chemotherapy-resistant cancerous tissues than in the sensitive cancerous tissues. Moreover, the results of ROC analysis revealed that the ASHGV40041402, ASHGV40002660, ASHGV40037204, ASHGV40000862, ASHGV40033167, ASHGV40021176, and ASHGV40033762 and the risk factor score had a powerful predictive ability. To sum up, the above lncRNAs had satisfactory prediction, and the risk factor score was also adapted to predict the FOLFOX chemotherapy response.

Several limitations need to be mentioned. First, the sample size was relatively small. We included mCRC patients who did not receive any treatment, which reduced the sample size. We intend to enlarge our sample size in the future. Second, the function and pathways of hub lncRNAs were conducted by lncRNAs microarray profiling and bioinformatics methods, and they should be further validated by experimental studies in the future.

In summary, the lncRNA expression of 11 mCRC patients receiving preoperative FOLFOX chemotherapy was analyzed by microarray analysis. The crucial functions enriched in chemotherapy-resistant modules were TNF signaling pathway and neutrophil-related immune regulation. Additionally, eight hub lncRNAs were identified and validated as new effective predictors for FOLFOX chemoresistance in mCRC patients and prognostic factors for non-metastatic CRC patients. Moreover, based on the hub lncRNAs, we constructed a risk factor model that had a strong power to predict FOLFOX chemoresistance and prognosis in CRC patients including non-metastatic and metastasis. These results may help to discriminate CRC patients who are candidates for FOLFOX chemotherapy. Nevertheless, more insightful molecular mechanisms are warranted in future studies.

## Data Availability Statement

The datasets presented in this study can be found in online repositories. The names of the repository/repositories and accession number(s) can be found at: https://www.ncbi.nlm.nih.gov/geo/, 138912.

## Ethics Statement

The studies involving human participants were reviewed and approved by this study was carried out in accordance with the committee of Fujian Medical University Union Hospital with written informed consent from all subjects. Written informed consent for participation was not required for this study in accordance with the national legislation and the institutional requirements.

## Author Contributions

YZ, MX, YS, ZX, and XL designed the experiments, performed the experiments, analyzed the data, and wrote the paper. YC and PC performed the experiments. All authors read and approved the final manuscript.

## Conflict of Interest

The authors declare that the research was conducted in the absence of any commercial or financial relationships that could be construed as a potential conflict of interest.

## Supplementary Material

Supplementary Figure 1 Cutoff points for hub lncRNAs ΔcT value determined by the X-tile program.

Supplementary Figure 2 The GSEA analysis of the eight lncRNAs.

Supplementary Table 1 Primer.

Supplementary Table 2 Baseline characteristics of CRC patients.
