# Supplementary figures and images for: Identification of LncRNAs Associated With FOLFOX Chemoresistance in mCRC and Construction of a Predictive Model
Source: Front Cell Dev Biol. 2021 Jan 28;8:609832. doi: 10.3389/fcell.2020.609832 (PMC7876414; doi:10.3389/fcell.2020.609832)

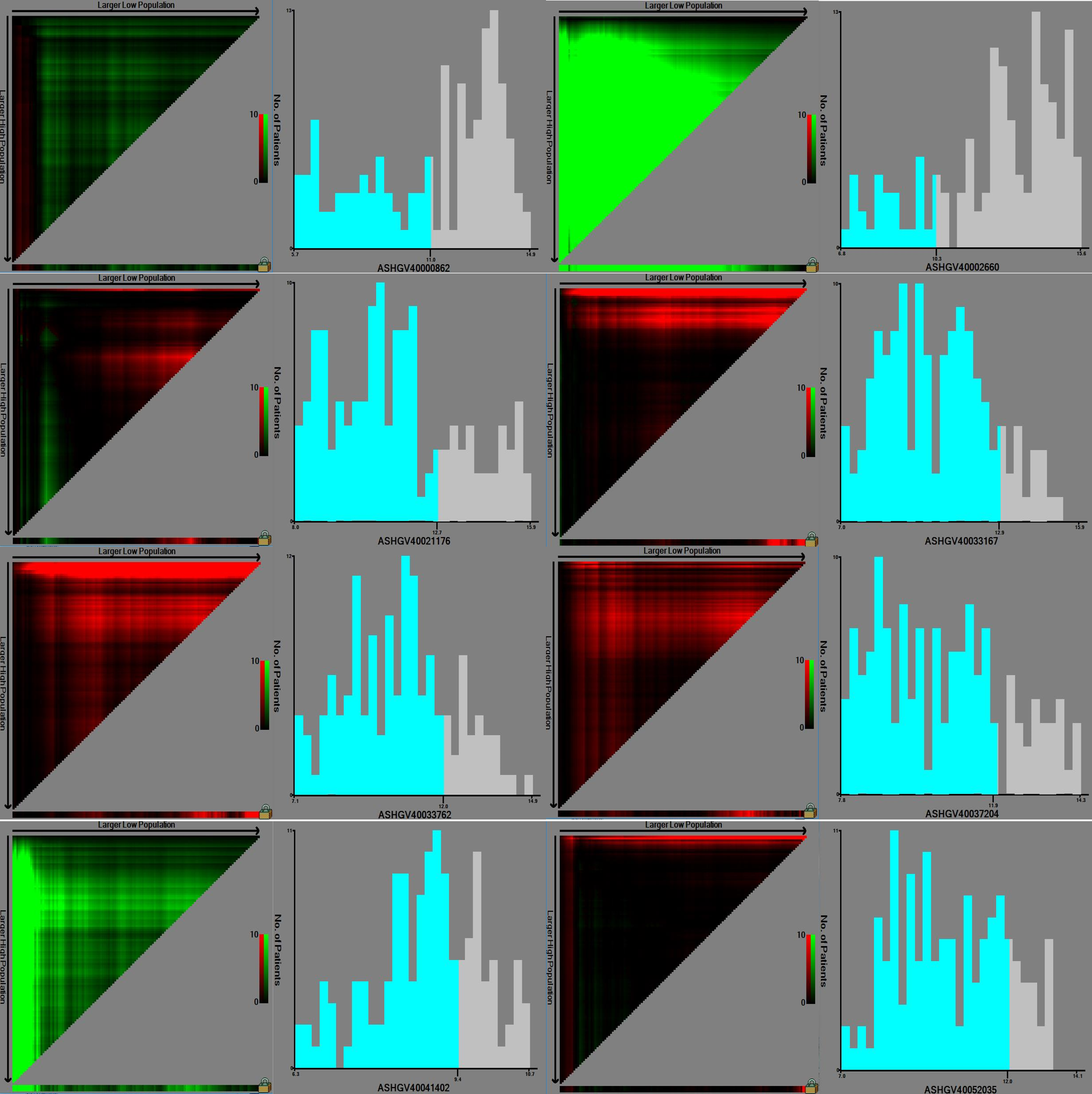

Supplement: Supplementary Figure 1 — Cutoff points for hub lncRNAs ΔcT value determined by the X-tile program. [file Image_1.TIF]

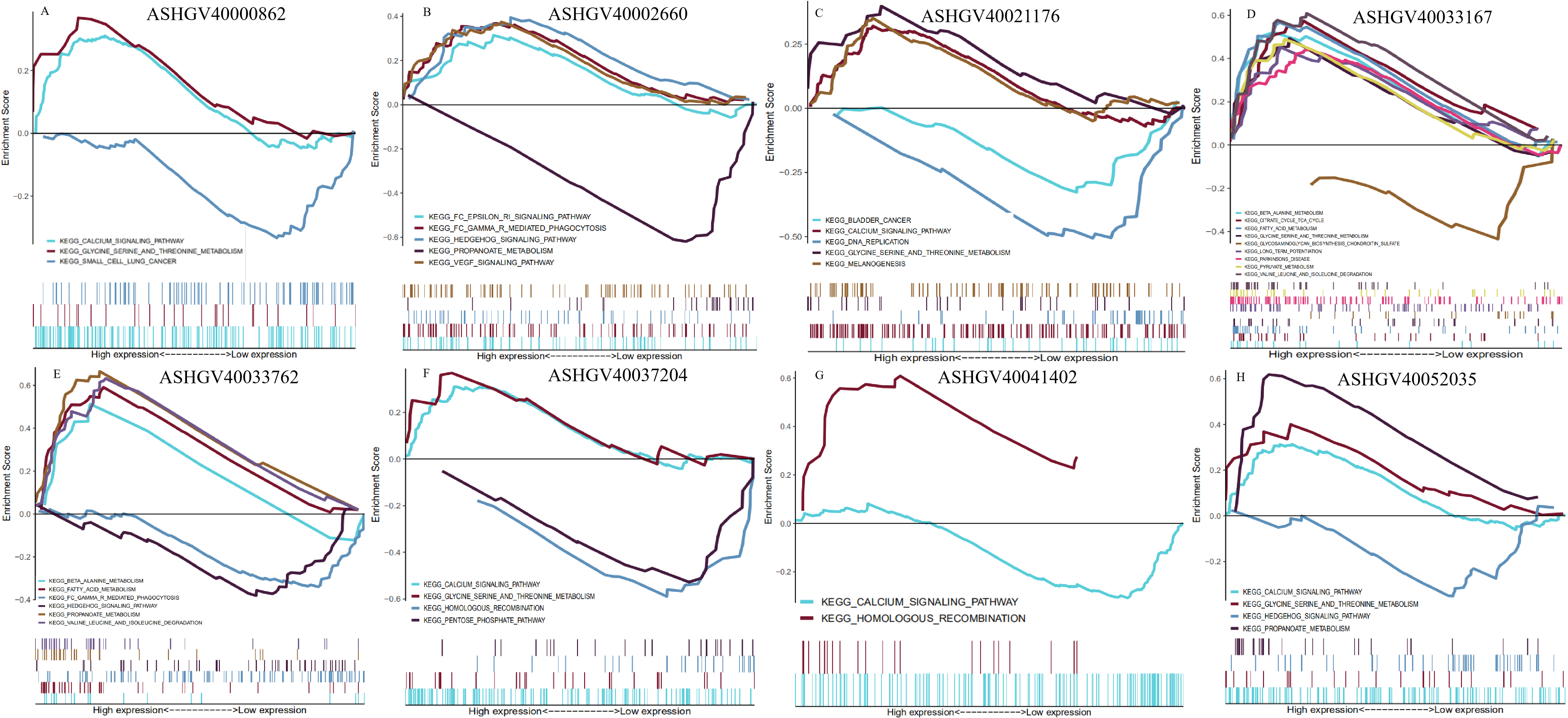

Supplement: Supplementary Figure 2 — The GSEA analysis of the eight lncRNAs. [file Image_2.TIF]
