# Supplementary material for: Identification of LncRNAs Associated With FOLFOX Chemoresistance in mCRC and Construction of a Predictive Model
Source: Front Cell Dev Biol. 2021 Jan 28;8:609832. doi: 10.3389/fcell.2020.609832 (PMC7876414; doi:10.3389/fcell.2020.609832)
Supplement: Supplementary Table 1 — Primer. [file Table_1.DOC]

| Gene | Forward | Reverse |
| --- | --- | --- |
| GAPDH | GGGAAACTGTGGCGTGAT | GAGTGGGTGTCGC TGTTGA |
| ASHGV40037204 | TAAGGGCTATGGGATTTGG | GCACGAGAATCGCTGAAAC |
| ASHGV40000862 | CAGGGACTATTTTAGGGGTTT | ACATTTCTGTGGAGGTTGACTC |
| ASHGV40041402 | CACAGTGAGGAAAGCCAGATG | TGGATTAGAATGCCATTGAGG |
| ASHGV40033167 | GCTAACACCATTCCCAACAC | CACCTTCATAGAGGGCTTCA |
| ASHGV40021176 | TGAATGCACCAAGGAAAGG | TCCAGGGCTACAGAAAACG |
| ASHGV40033762 | CCTTGGAACCTCCTGAAGC | GGTCTGTAATTCTCAAGCCCTA |
| ASHGV40052035 | GCGAGGTGAATGAAGGACA | TACTCAGGAGGCTGAGGGAG |

Supply Table1 Primer
