# Supplementary material for: Identification of LncRNAs Associated With FOLFOX Chemoresistance in mCRC and Construction of a Predictive Model
Source: Front Cell Dev Biol. 2021 Jan 28;8:609832. doi: 10.3389/fcell.2020.609832 (PMC7876414; doi:10.3389/fcell.2020.609832)
Supplement: Supplementary Table 2 — Baseline characteristics of CRC patients. [file Table_2.doc]

**Supply Table 2** Baseline characteristics of CRC patients

| **Characteristics** | **Non-metastasis CRC patients**  **(n=136)** | **mCRC patients**  **(n=73)** |
| --- | --- | --- |
| Sex (%) |  |  |
| Male | 81 (59.6) | 48(65.8) |
| Female | 55 (40.4) | 25 (34.2) |
| Age (years) | 61.1 ± 11.8 | 58.4± 12.9 |
| Response to the neo-chemotherapy |  |  |
| PR | - | 25(34.2) |
| SD | - | 20 (27.4) |
| PD | - | 28 (38.4) |
| ASA score (%) |  |  |
| 1 | 72 (73.5) | 27 (37.0) |
| 2 | 16 (16.3) | 39 (53.4) |
| 3 | 10 (10.2) | 6 (8.2) |
| BMI | 22.2±3.3 | 22.6± 2.7 |
| Tumor location |  |  |
| Ascending colon | 24 (17.6) | 6 (8.2) |
| Transverse colon | 15 (11.0) | 5(6.8) |
| Descending colon | 12 (8.8) | 2 (2.7) |
| Sigmoid colon | 33 (24.3) | 11 (15.1) |
| Rectum | 52 (38.2) | 49 (67.1) |
| CEA level (%) |  |  |
| <5.0 ng/ml | 82 (60.3) | 25(34.2) |
| ≥5.0 ng/ml | 54(39.7) | 28 (38.4) |
| Unknown | 0(0) | 20(27.4) |
| CA19-9 level (%) |  |  |
| <37.0 U/ml | 120(88.2) | 40 (54.8) |
| ≥37.0 U/ml | 15 (11.8) | 13 (17.8) |
| Unknown | 0(0) | 20(27.4) |
| Histopathology |  |  |
| Ulcering | 79 (58.1) | 46(73.0) |
| Expanding | 56 (41.2) | 26(35.6) |
| Infiltrating | 1 (0.7) | 1 (1.4) |
| Histopathology (%) |  |  |
| Adenocarcinoma | 106 (77.9) | 67 (91.8) |
| Mucinous or signet ring cell carcinoma | 30 (22.1) | 6 (8.2) |
| Tumor differentiation (%) |  |  |
| Well/ moderately differentiated | 101 (74.2) | 63 (86.3) |
| Poorly differentiated and others | 35 (25.8) | 10 (13.7) |
| Postoperative hospital stay (days) | 8.3 ± 6.3 | 8.7± 7.9 |
| Tumor size (cm) | 4.6 ± 2.2 | 4.3 ± 2.2 |
| Pathological T stage (%) |  |  |
| 1 | 5(3.7) | 0 (0) |
| 2 | 27 (19.9) | 13 (17.8) |
| 3 | 93 (68.4) | 47(64.4) |
| 4 | 11 (8.1) | 13 (17.8) |
| Pathological N stage (%) |  |  |
| N0 | 88 (64.7) | 32(43.8) |
| N1 | 31 (22.8) | 22 (30.1) |
| N2 | 17 (12.5) | 19 (26.0) |
| Nerval invasion (%) | 32 (23.5) | 18 (24.7) |
| Vascular invasion (%) | 11 (8.1) | 11 (15.1) |

ASA, American Society of Anesthesiologists; BMI, Body Mass Index; CEA, Carcinoma Embryonic Antigen; CA19-9, Carbohydrate Antigen19-9; CRC, Colorectal Cancer; mCRC, Metastasis CRC;
